# A fertile future: Fertility preservation special series

**DOI:** 10.1530/RAF-22-0001

**Published:** 2022-02-11

**Authors:** Rod T Mitchell, Suzannah A Williams

**Affiliations:** 1 MRC Centre for Reproductive Health, The Queen’s Medical Research Institute, The University of Edinburgh, Edinburgh, UK; 2 Department of Paediatric Endocrinology, Royal Hospital for Children and Young People, Edinburgh, UK; 3 Nuffield Department of Women’s and Reproductive Health, Women’s Centre, John Radcliffe Hospital, University of Oxford, Oxford, UK

## Abstract

Fertility preservation is a rapidly advancing field with numerous broad applications ranging from retaining the prospect of fertility in a child with cancer to protecting an entire species from extinction. In recent years, huge strides have been made in understanding the biology of male and female reproduction in animals and humans and using this knowledge to develop strategies for fertility preservation across a range of clinical and ecological applications. This *Reproduction and Fertility* preservation series is composed of articles from experts on this topic and these will highlight key developments in fertility preservation and also identify the challenges that still face this exciting and relatively new field.

## Introduction

Fertility preservation is a rapidly advancing field with numerous broad applications ranging from retaining the prospect of fertility in a child with cancer to protecting an entire species from extinction. In recent years, huge strides have been made in understanding the biology of male and female reproduction in animals and humans and using this knowledge to develop strategies for fertility preservation across a range of clinical and ecological applications. This *Reproduction and Fertility* preservation series is composed of articles from experts on this topic: these will highlight key developments in fertility preservation and also identify the challenges that still face this exciting and relatively new field. Several leading authors in the field will be contributing, however, as this series is being published by *Reproduction and Fertility*, it is online and open access, and there is scope for more contributions. So do get in touch if you have something to enhance this excellent series in fertility preservation.

## *A Tale of Two Testes:* Fertility preservation in males

When it comes to offering fertility preservation in males at risk of infertility, a key question to be asked is *what are we trying to preserve?* While preserving germ cells is key, the approach to fertility preservation will depend on which germ cells are present – that is does the tissue contain sperm or not? Given the fundamental differences between the prepubertal and adult testis, consideration of age, pubertal stage and maturity are paramount when thinking about the options to preserve male fertility.

For adult males who face gonadotoxic treatments, cryopreservation of sperm containing semen is a proven option that will preserve the potential to father biological children, albeit with a requirement for assisted reproductive technologies (ARTs), which in its simplest form is intrauterine insemination or, the more involved,* in vitro* fertilisation or intracytoplasmic sperm injection. Freezing semen should be offered to all men due to receive any treatment with a risk of impaired fertility (e.g. chemotherapy or radiotherapy). But what about when spermatogenesis is impaired? In this series, Dr Sarah Martins Da Silva will discuss the options and practicalities in this challenging situation.

Adolescence represents a difficult period when it comes to fertility preservation in males. Physical and emotional maturity of the patient plays a key role in what can be offered and important ethical considerations apply, for example, does the patient have the capacity to consent for treatment? Semen cryopreservation may be an option if the individual is well established in puberty, while surgical intervention to extract sperm can also be considered. However, for those whose testes do not yet make sperm (e.g. children), fertility preservation must focus on preserving the sperm precursor cells, the spermatogonial stem cells (SSC), in the tissue by cryopreservation of testicular tissue. While animal studies involving re-implantation of cryopreserved testicular tissues provide great promise for the future with sperm growing in the transplants (Fayomi *et al*. 2019), we still await the first report of successful restoration of fertility and livebirth in humans using these cryopreserved tissues. For children, this approach to obtain a testicular biopsy to preserve SSCs has recently been established in a limited number of centres worldwide and remains the only option for fertility preservation in this age group (Goossens *et al*. 2020). Identifying those patients at risk who may benefit from testis tissue cryopreservation is key and Dr Sheila Lane will discuss patient eligibility and options for fertility preservation in this context. While such cryopreservation programmes can store tissues, research is required to focus on ensuring the freezing process maintains viability and function. In addition, the options to restore fertility potential with tissue/spermatogonial stem cell transplantation or* in vitro* spermatogenesis must be translated from existing animal studies. Dr Jan-Bernd Stukenborg will describe how temperature may play a key role in this regard.

## *Eggvolution*: Fertility preservation in females

The potential for fertility preservation in females differs from males but is also dependent on age.

For post-pubertal females who are due to undergo gonadotoxic treatment, such as chemotherapies, if there is time, the option exists to undergo a cycle of hormonal stimulation to enable egg collection. These eggs can be stored frozen or fertilised and the resultant embryos are frozen. However, careful consideration must be given to the maturity of the patient since egg collection is invasive requiring intravaginal ultrasound. Therefore, although it is possible to harvest eggs from post-pubertal teenagers, this procedure is usually restricted to females over the age of 18 years of age. For girls and teenagers, the only real option is ovarian tissue cryopreservation. The options for use of this tissue are also diverse and will be discussed in this series. For this group of patients, the aim is to re-implant the cryopreserved ovarian tissue once the patient has reached adulthood and wishes to start a family. Although success has recently been achieved for women whose tissue was cryopreserved in childhood, with the live birth of babies post-re-implantation (Dolmans *et al*. 2021), there is clearly much to be done to establish this technique. However, for patients with blood cancer, the tissue is not routinely re-implanted due to the potential for reintroducing cancer, and therefore *in vitro*culture of the ovarian tissue to generate mature eggs is required. However, such methodology is still being optimised and is far from clinical treatment and will be reviewed by Dr Stine Kristensen. Clearly, a lot of work is required in this field to achieve start-to-finish fertility preservation for young girls and work into generating artificial ovaries to assist in this aim is ongoing. Finally, in an effort to bypass the damage caused by chemotherapy drugs altogether, research into ways of preserving ovarian function during such regimens is ongoing andProf Karla Hutt and Dr Roseanne Rosariowill be contributing articles to the series on this subject.

## *To Do or Not to Do:* Making an informed choice for fertility preservation

The first step on the path towards the provision of fertility preservation is awareness of the available clinical options, which should be explored for those facing infertility where appropriate. However, access to fertility preservation services and utilisation by clinical teams across the UK is not consistent (Newton *et al*. 2021). Currently in the UK, fertility preservation involving gametes is conducted within local fertility centres, while a centralised service for ovarian and testicular tissue cryopreservation for children and young adults is available in Edinburgh and Oxford. However, ensuring that all those undergoing gonadotoxic treatment are appropriately counselled and referred to such services before their treatment remains a priority. Once a patient arrives in the clinic, the decisions made are important and potentially life altering. Providing accurate and readily accessible information to facilitate decision making is crucial for patients at this time and huge strides have been made in this field by Professor Georgina Jones*,*as described in her review in this series.

## *It’s not too late:* fertility preservation for preventing species extinction

Fertility preservation in the form of semen cryopreservation has been used as a tool in agriculture for decades to enable the genetics of individual prize bulls to be spread far and wide. This tool and more recently the more complex ARTs have been utilised in order to preserve endangered species. Professor Thomas Hildebrandtleads the Biorescue programme which has been focused on preventing the extinction of the Northern White Rhino – just two females remain. Prof Hildebrandt and his team will be contributing a review describing the current progress and future of ARTs in preserving endangered species. The use of biobanking by cryopreservation of germ cells, gonads and tissues from both males and females to prevent the loss of endangered species is also key to retaining diversity on this planet. We have already lost numerous species of rhino on this planet in recent years, including the Western Black Rhino, and worldwide efforts are ongoing to prevent the loss of such iconic species. Biobanking and its role in preventing extinction will be discussed in a review led by Dr Rhiannon Bolton, the coordinator of the UK charity Nature’s SAFE (Saving Animals From Extinction). Despite the reliability of cryopreservation in preserving fertility, new techniques are being developed and will be discussed by Professor Pierre Comizzoli.

## Fertility preservation for all: preserving fertility in under-represented groups

Successful development of multiple fertility preservation options for indications such as prior to gonadotoxic treatments has lead clinicians and researchers to explore options in other patient groups such as those with disorders (or differences) in sex development (Islam *et al*. 2019). Challenges exist when applying fertility preservation in this context and the options available are largely dependent on the underlying condition and the gonadal phenotype of the individual (Gomes *et al*. 2020). The transgender community is another important group where offering fertility preservation for individuals is increasingly recognised and *Dr James Barrett* will discuss the options and challenges in this context.

## Mind the gaps: planning for the future of fertility preservation

This series of articles focusing on fertility preservation for patients and conservation of endangered species will, we hope, not only educate but also stimulate discussion, and challenge ideas and identify barriers with the aim of working towards ensuring fertility preservation is available for all where required. As the series aims to highlight areas in the field of fertility preservation that require attention or focus, authors of studies that may fit within this *Reproduction and Fertility* series are encouraged to get in contact. We hope you enjoy reading the articles in this series.

## Declaration of interest

Suzannah Williams is an Associate Editor and a Lay Editor of *Reproduction and Fertility*. Suzannah Williams was not involved in the review or editorial process for this paper, on which she is listed as an author.

## Funding

R T M is supported by UKRI Future Leaders Fellowship (MR/S017151/1). The MRC Centre for Reproductive Health at the University of Edinburgh is supported by MRC (MR/N022556/1).
